# Presacral dermoid cyst in a young female patient: A case report

**DOI:** 10.1002/ccr3.5062

**Published:** 2021-11-11

**Authors:** Diptee Poudel, Bibek Man Shrestha, Bishnu Prasad Kandel, Suraj Shrestha, Ankush Kansal, Paleswan Joshi Lakhey

**Affiliations:** ^1^ Maharajgunj Medical Campus, Institute of Medicine Maharajgunj Nepal; ^2^ Department of Surgical Gastroenterology Tribhuvan University Teaching Hospital Maharajgunj Nepal

**Keywords:** dermoid cyst, laparoscopy, presacral, retrorectal

## Abstract

Presacral dermoid cysts are rare, benign tumors of developmental origin that primarily affect women. Surgical resection is the mainstay of treatment for these cysts, even if asymptomatic, with the laparoscopic approach being safer and more efficient.

## Graphical abstract

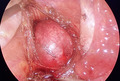

## 1 INTRODUCTION

Presacral tumors are a rare and heterogeneous group of tumors occurring in the potential space between the rectum and the sacrum. Their true incidence in the general population is not known; however, they are estimated to account for about 1 in 40,000 hospital admissions.^1^ The median age at diagnosis of the disease varies across studies from 40 to 50 years, with women being the most affected.^1^, ^2^, ^3^, ^4^

Presacral tumors range from simple benign cysts to complex malignant masses, with dermoid cysts accounting for fewer than 5% of the tumors.^1^, ^2^ Herein, we report a case of a female patient diagnosed with a presacral dermoid cyst that was resected via a laparoscopic approach.

## 2 CASE PRESENTATION

A 22‐year‐old female patient presented to our center with a chief complaint of insidious‐onset, continuous, and non‐radiating lower back pain for the past 6 months. She had no history of nausea, vomiting, fever, hematochezia/melena, abdominal distension, weight loss, or trauma. Her bowel and bladder habits were normal, and she had no significant past medical history or family history of malignancy.

On examination, the patient was afebrile, hemodynamically stable, and fully conscious. Her abdomen was soft and non‐tender with normal bowel sounds, and digital rectal examination revealed no abnormalities. Examination of all other systems was normal. Blood counts were within normal ranges, and liver and renal function tests were unremarkable.

**FIGURE 1:**
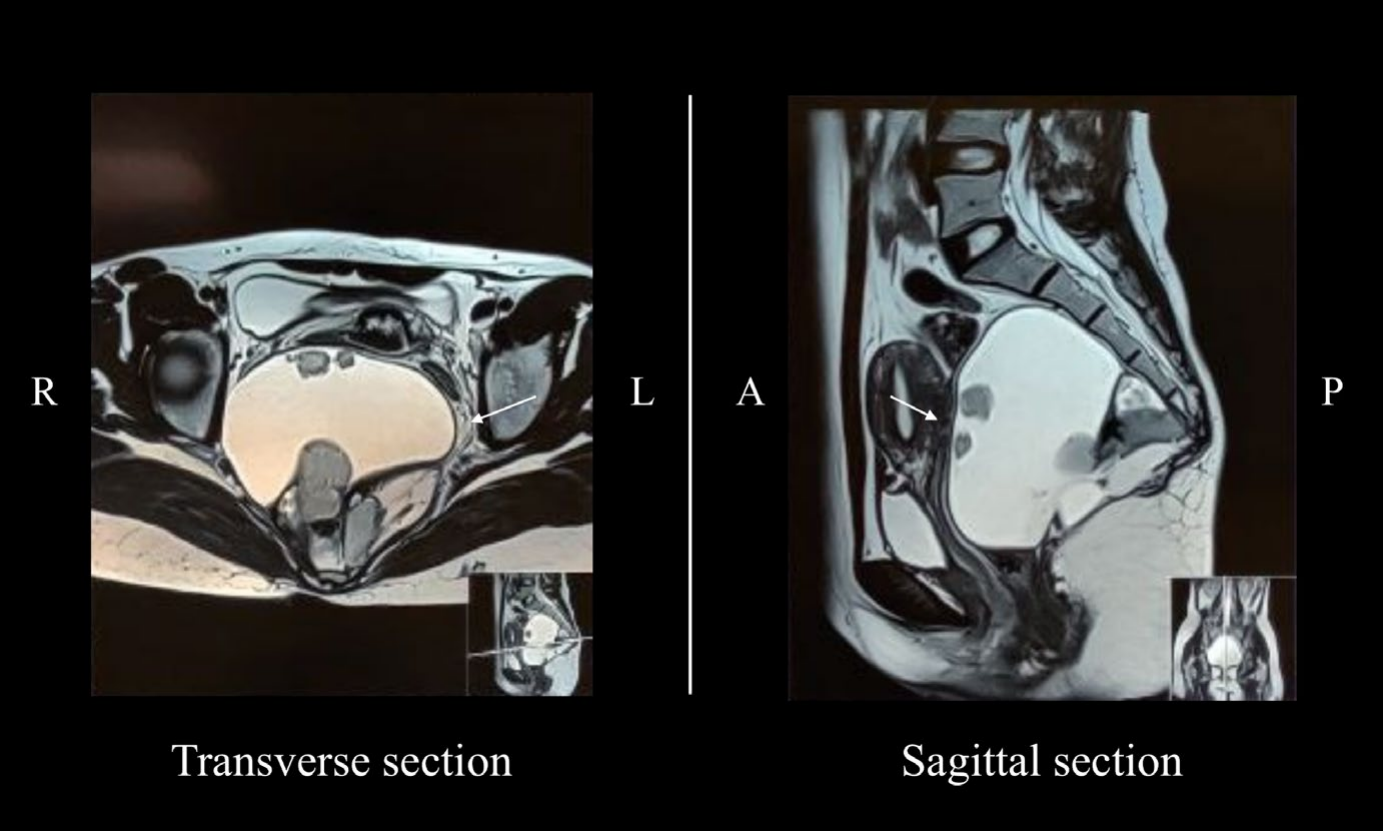
Magnetic resonance imaging of the pelvis showing a presacral lesion

Transabdominal ultrasonography of the pelvis showed a complex cystic lesion with dimensions of 10.2 cm × 6.4 cm. A subsequent magnetic resonance imaging (MRI) scan revealed a 10.6 cm × 10 cm × 9.4 cm sized multiloculated cystic lesion in the presacral space with variable signal intensities, giving an impression of a type IV sacrococcygeal teratoma. (Figure 1).

**FIGURE 2:**
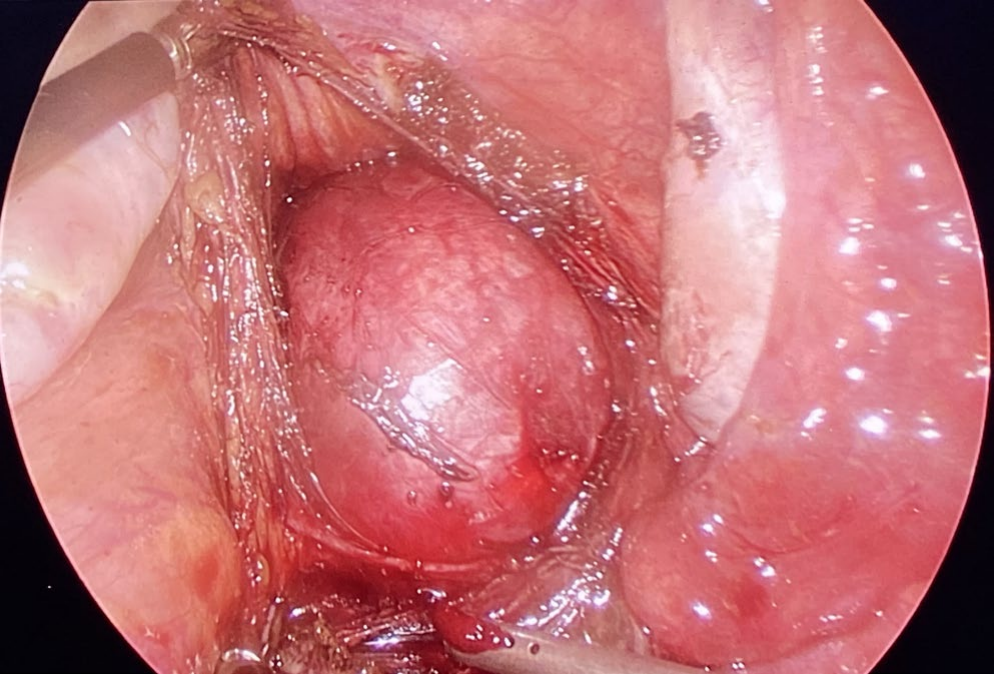
Laparoscopic view of the dermoid cyst with its adhesions

**FIGURE 3:**
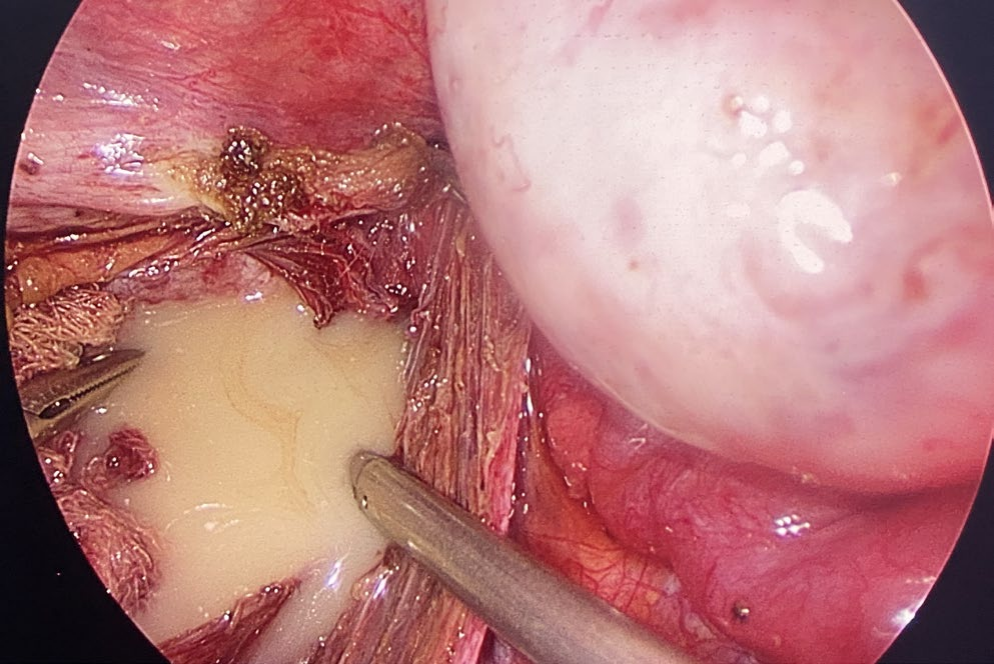
Ruptured cyst with its purulent content

**FIGURE 4:**
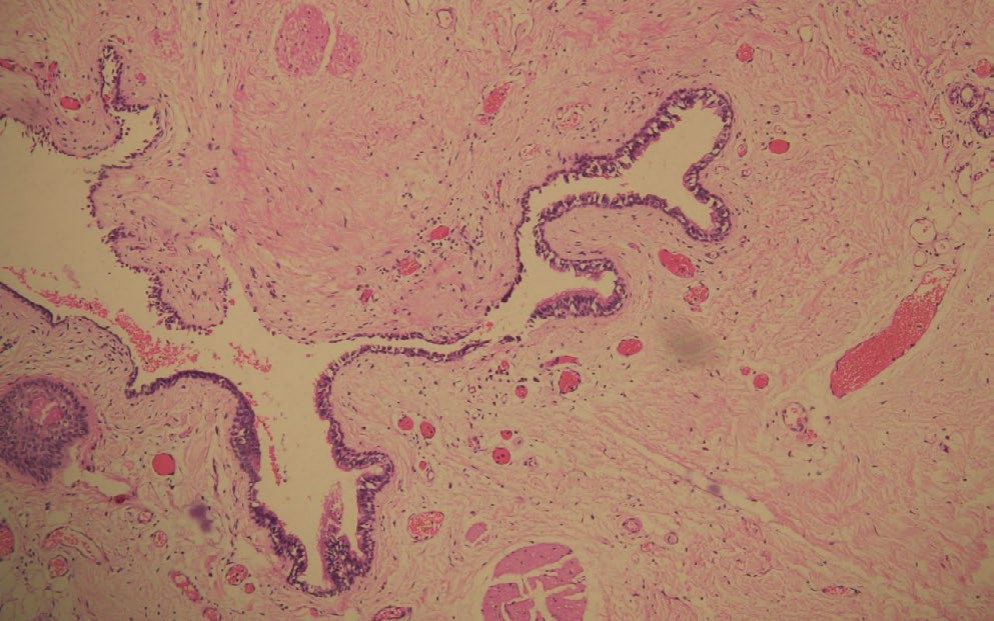
Section showing a cyst lining of squamous epithelium with foci of pseudostratified ciliated columnar epithelium, along with eccrine ducts and hair follicles in the underlying stroma (H&E, ×200)

The patient eventually underwent a laparoscopic excision of the cyst. Preoperatively, a cyst about 10 cm × 10 cm in size occupying the presacral space was observed, adherent to the levator ani muscles and the coccyx and pushing the rectum anteriorly and to the left. (Figure 2) Accidental rupture of the cyst occurred during adhesiolysis releasing its purulent content that was immediately drained, followed by a thorough cleaning of the region with warm normal saline. (Figure 3) Histopathological examination of the resected mass revealed a cyst lining of squamous epithelium with foci of pseudostratified ciliated columnar epithelium. Skin adnexa like eccrine ducts and hair follicles could be observed in the underlying stroma. (Figure 4).

The patient made an uneventful recovery following the surgery and was discharged on the 4th postoperative day. She was stable at the time of discharge, and her abdominal pain had significantly resolved. She is doing well and remains disease‐free at 6 months of follow‐up.

## 3 DISCUSSION

The presacral or retrorectal space is bounded anteriorly by the rectum, posteriorly by the sacrum and coccyx, superiorly by the peritoneal reflection, inferiorly by the levator ani and coccygeus muscles, and laterally by the ureters and iliac vessels. The space can lodge a group of rare and heterogeneous tumors that may be solid or cystic, with the solid variety having a higher malignancy rate. The tumors become symptomatic due to their mass effect on the surrounding organs and nerves, and patients present with nonspecific symptoms like pelvic pain or pressure, change in bowel habits, urinary or fecal incontinence, and sexual dysfunction. However, many patients with presacral tumors are asymptomatic, and the lesions are discovered incidentally, either at routine physical examination or at imaging for an unrelated indication.^5^, ^6^

Presacral dermoid cysts are rare, benign tumors of developmental origin that primarily affect women. They can be uni‐ or multilocular, with content varying from clear fluid to dense mucus. Histologically, their cyst wall is lined with more than one type of epithelium, which can be squamous, respiratory, or gastrointestinal, and consists of skin appendages like sweat glands, hair follicles, or sebaceous cysts in the underlying stroma.^5^

It is difficult to diagnose a presacral dermoid cyst by ultrasonography because their proximity to the ovary can lead to a misdiagnosis of an ovarian cyst. Thus, CT/MRI of the pelvis must be considered if a presacral mass is suspected. CT scan shows the cyst as a presacral hypodense lesion with well‐demarcated edges, while on MRI, the content of the cyst determines its signal intensity. Adjacent visceral invasion seen in imaging would suggest malignant changes. A preoperative biopsy is usually contraindicated for any presacral lesion that is surgically resectable because of the risk of infecting the lesion and seeding the biopsy tract with malignant cells in the case of a malignant lesion.^5^, ^6^

Surgical resection is the standard treatment modality for presacral tumors, even if they are asymptomatic, as they might harbor malignancy, become infected, or in the case of teratomas, undergo malignant degeneration. There are three common approaches for the resection of these tumors—anterior (transabdominal), posterior (perineal), and combined abdominoperineal. Accurate diagnostic imaging is essential in evaluating the location and size of these tumors and their relationship with the adjacent structures, which help determine the surgical approach. Tumors that extend above the level of the S4 vertebra can be removed either through the anterior or combined approach, while for low‐lying lesions, the posterior approach is indicated.^5^, ^6^ The tumors can be resected using a laparoscope to avoid the considerable morbidity associated with open surgery.^7^, ^8^, ^9^, ^10^ As postoperative fistula can occur in up to 3% of the cases, careful dissection away from the rectum must be stressed.^11^ Recurrence rates of benign presacral lesions are meager compared to malignant tumors; however, the rates can vary depending on the extent of resection.^5^

## 4 CONCLUSION

The rare possibility of presacral tumors must be considered in women with suggestive symptoms, which can only be ruled out by accurate pelvic imaging. Surgery is the mainstay of treatment for these tumors, with the laparoscopic approach being safer and more efficient as it reduces surgical trauma and offers excellent visualization of the deep structures in the presacral space.

## CONFLICTS OF INTEREST

The authors declare that they have no competing interests.

## AUTHOR CONTRIBUTIONS

Diptee Poudel, Bibek Man Shrestha, and Suraj Shrestha wrote the original draft of the manuscript. All authors contributed to the review and editing of the manuscript. Ankush Kansal, Bishnu Prasad Kandel, and Paleswan Joshi Lakhey were involved in the diagnosis and treatment of the patient. All authors have read and approved the final version of the manuscript.

## CONSENT

Written informed consent was obtained from the patient for publication of this case report and any accompanying images.

## Data Availability

All the necessary information is provided within the manuscript.
